# Multicomponent Supportive-Educative mHealth Intervention for Self-Care Among Individuals With Osteoporosis in Thailand: Exploratory Pretest-Posttest Pilot Study

**DOI:** 10.2196/101025

**Published:** 2026-08-20

**Authors:** Natthida Pangutha, Sirinapha Jittimanee, Porramat Saksaen

**Affiliations:** 1 Faculty of Nursing, Chulalongkorn University, Pathumwan, Bangkok, 10330, Thailand; 2 Center of Excellence for Enhancing Well-being in Vulnerable and Chronic Illness Populations, Faculty of Nursing, Chulalongkorn University, 254 Rama 1 Road, Wangmai Subdistrict, Pathumwan DistrictBangkok, 10330, Thailand, +6622181377; 3 Chaiprakan Hospital, Ministry of Public Health, Chaiprakan district, Chiang Mai province, 50320, Thailand

**Keywords:** osteoporosis, self-care, telemedicine, family support, exercise, accident prevention

## Abstract

**Background:**

Poor self-care impairs osteoporosis management. Disease-related knowledge and family support have influenced self-care; however, the combined association of these factors with self-care remains insufficiently understood.

**Objective:**

This study aimed to compare self-care among individuals with osteoporosis who received a multicomponent supportive-educative mobile health intervention with that of individuals who received usual care.

**Methods:**

This exploratory pilot study using a pretest-posttest design with a nonequivalent control group included 46 individuals aged 50 years and older who had been diagnosed with osteoporosis and were receiving care at the orthopedic outpatient department of a university hospital in Bangkok. Participants were matched by sex, age, and back pain scores and then sequentially allocated to an intervention group (n=23, 50%) and a comparison group (n=23, 50%). The intervention group received a 4-week multicomponent supportive-educative intervention based on Orem’s self-care theory, delivered partly through a closed Facebook group. The intervention included educational guidance, video-based teaching, psychological support, family involvement, and remote nurse interaction. The comparison group received usual care. Self-care was measured using the Self-Care of Osteoporosis Scale. Chi-square tests, Fisher exact tests, independent-sample 2-tailed *t* tests, analysis of covariance, and multivariate analysis of covariance were used to examine differences between groups.

**Results:**

After controlling for baseline self-care scores, participants in the intervention group had significantly higher postintervention self-care scores than those in the comparison group (*F*_1,43_=459.29; *P*<.001). Significant group differences were also observed across all 3 domains of self-care after controlling for baseline scores. Compared with the control group, the intervention group demonstrated significantly higher postintervention scores in diet and physical activity (*F*_1,41_=245.04; *P*<.001), safety performance (*F*_1,41_=171.20; *P*<.001), and medication adherence (*F*_1,41_=144.54; *P*<.001).

**Conclusions:**

The multicomponent supportive-educative intervention delivered partly through Facebook was associated with improvements in self-care among individuals with osteoporosis. Future rigorous trials are needed to confirm its efficacy.

## Introduction

Osteoporosis affects 23.1% of older women and 11.7% of older men worldwide [1]. It is associated with increased risk of osteoporotic fractures, particularly at the femoral neck (31.9%), spine (28.8%), and hip (9.9%) [2]. The causes of osteoporosis include reduced estrogen levels after menopause, inadequate intake of vitamin D and calcium, the use of certain medications (eg, anticoagulants), and certain diseases (metabolic and hormonal disorders) [3]. Effective management of osteoporosis relies on both medical treatment and individuals’ engagement in self-care, including medication adherence, regular exercise, adequate dietary intake, and fall prevention, to reduce the risk of complications.

Self-care among individuals with osteoporosis remains a significant challenge. One study reported that 100% of women with osteoporosis did not engage in exercise, whereas mean daily calcium and vitamin D intake was 546.7 (SD 60.28) mg and 2.0 (SD 0.34) µg, respectively [4]. Both values were below the recommended daily intake of 1000 to 1200 mg for calcium and 15 to 20 µg for vitamin D [5]. These behaviors include discontinuing medication without medical consultation [6]. Self-care for osteoporosis refers to behaviors undertaken to manage the condition and prevent complications. Nurses play a vital role in supporting and encouraging individuals with osteoporosis to adopt these self-care practices [7].

Past research has identified several factors influencing self-care in individuals with osteoporosis, including disease-related knowledge, social support, patient–health care provider relationship, medication-related side effects, and patient participation in treatment. In one study, poor disease-related knowledge was reported in 52.8% of patients and was associated with inadequate intake of vitamin D and calcium [8]. Social support and a strong patient-physician relationship have been positively associated with self-care behaviors [9]. Medication-related side effects, including those associated with self-administered Forteo, also influence medication self-care [6]. In addition, patient participation in treatment decisions has been linked to improved treatment outcomes [10].

Interventions to promote self-care among individuals with osteoporosis have been widely studied. These include educational video-based programs, group training sessions, telephone follow-ups, and support from health care teams. There is evidence suggesting that such interventions significantly improve medication adherence and reduce the risk of fractures [11,12]. However, most of the previous interventions focus primarily on individuals (participants engaged in the interventions themselves without the involvement of family members) even though many are older adults who may require assistance from family members and may find it difficult to follow instructions independently. Integrating family support with the supportive-educative nursing system by Orem et al [13], which emphasizes teaching, guidance, emotional support, and the creation of an environment conducive to self-care, may better address these needs and enhance self-care among individuals with osteoporosis.

In Thailand, 1 in 5 women aged 40 to 80 years has osteoporosis, and the prevalence exceeds 50% among those aged 70 years and above [14]. However, challenges similar to those in other countries persist; only 67.2% of individuals have adequate calcium intake, and overall levels of physical activity remain low [15]. These problems highlight the need for interventions to promote self-care among individuals with osteoporosis. With the rapid advancement of mobile health (mHealth) technologies and the widespread use of social media, these platforms offer promising opportunities for delivering supportive-educative interventions and promoting healthy behaviors, as reported in previous studies [16]. Family support also plays a significant role in enhancing self-care, as shown in previous studies [17]. Addressing this gap is crucial for enhancing self-care among individuals with osteoporosis. Therefore, this study aimed to compare self-care between individuals with osteoporosis who received a supportive-educative mHealth intervention and those who received usual care. We hypothesized that participants receiving the multicomponent supportive-educative mHealth intervention would demonstrate significantly higher overall self-care mean scores and higher individual mean scores in the 3 domains of self-care (medication adherence, diet and physical activity, and safety performance) than those receiving usual care.

## Methods

### Study Design

This exploratory pilot study used a pretest-posttest design with a nonequivalent control group. The study followed the TREND (Transparent Reporting of Evaluations With Nonrandomized Designs) checklist [18].

### Study Setting

This study was conducted at an outpatient clinic in a 1200-bed university hospital in Bangkok. In 2025, a total of 256 individuals with osteoporosis received care at this hospital, where osteoporosis services are provided at the orthopedic outpatient department every Wednesday. Medical appointments are scheduled every 4 to 6 weeks.

### Participants

#### Eligibility Criteria

Participants were individuals diagnosed with osteoporosis. Eligibility criteria were (1) age of 50 years or older, (2) receipt of treatment for 6 months or more, (3) residence with family members or relatives, and (4) either use of Facebook or having a family member who used Facebook. Individuals were excluded if they were severely ill; were scheduled for surgery during the intervention period; had speech or hearing impairments; or had cognitive impairment, defined as a score of 22 or lower on the Thai version of the Mini-Mental State Examination.

The sample size was calculated using an effect size of 0.8, a significance level of .05, a power of 0.80, and a *t* test, resulting in a required sample size of 21 individuals per group. Allowing for a 10% dropout rate, the final sample size was 23 participants per group.

#### Method of Recruitment

Eligible participants were consecutively identified by nurses at the time of clinic check-in. Nurses provided a flyer to invite them to participate in the study. Interested individuals were referred to the researcher, who explained the study procedures. During the enrollment period between July 2025 and August 2025, a total of 120 individuals with osteoporosis attended the clinic, of whom 88 (73.3%) were eligible and invited, and 46 (52.3%) agreed to participate. All individuals who declined participation were female. Participants were matched 1:1 based on sex, age, and back pain score because these variables influenced self-care [6,9].

#### Assignment Method

Participants were assigned alternately to the intervention and comparison groups in a 1:1 ratio. This assignment method was used to maintain comparable group sizes and facilitate group matching based on key participant characteristics.

#### Blinding

Due to the nature of the multicomponent supportive-educative mHealth intervention, blinding of participants and researchers was not feasible. Participants were aware of their group assignment, and the researchers delivering the intervention were not blinded.

### Usual Care

During the study period, the comparison group continued to receive usual care. Nurses provided health education focusing on medication, diet, and exercise. This education was delivered orally, without the use of written materials or video clips for exercise demonstration. Individuals could confirm or reschedule their appointments through the hospital application.

### Intervention Program

#### Intervention Description

The multicomponent intervention was delivered through a private Facebook group over a 4-week period (28 days). It consisted of 4 weekly 15-minute educational sessions conducted in small groups of 3 to 4 participants. Live Facebook sessions were held daily between 3 PM and 4 PM to accommodate participants’ schedules. Each participant and 1 family member attended 1 session per week using a shared study-specific Facebook account. Participants who missed their scheduled session received a reminder from the researcher and attended a subsequent session. Outcome assessments of self-care were conducted at 2 time points: baseline and 1 week after completion of the intervention during face-to-face visits at the outpatient department.

The multicomponent intervention focused on three domains of self-care: (1) medication adherence, (2) diet and physical activity, and (3) safety performance. It was developed based on Orem’s self-care theory [13], specifically the supportive-educative nursing system, and incorporated the helping methods by Orem et al [13] by (1) guiding and directing participants to strengthen self-care ability and decision-making; (2) teaching through four 10-minute educational video clips covering osteoporosis and medication, nutrition, exercise, and fall prevention; (3) providing psychological support through online family reflection sessions; and (4) creating a supportive learning environment through regular nurse-participant interactions (Multimedia Appendix 1). A printed handbook containing the same educational content as the 4 video clips was provided to reinforce learning throughout the intervention. An overview of the intervention is shown in Table 1.

**Table 1.** Intervention overview.

	Objectives	Key activities	Delivery method	Helping methods by Orem et al [13]	Monitoring and evaluation
Session 1 (baseline)	Establish relationships with individuals and families	Introduce the intervention Help participants and families share self-care experiences Assess baseline self-care and back pain scores using an interviewer-administered questionnaire Provide self-care handbook	Face-to-face	Guiding and directing	Baseline self-care
Session 2 (week 1)	Explain disease management	Learn about osteoporosis, medication, nutrition, and exercise Upload four 10-min video clips, which are available for repeated viewing Participate in questions and answers	Online group session via closed Facebook group	Teaching	Ability to identify content from the video and handbook
Session 3 (week 2)	Reflect on self-care experience	Facilitate the sharing of experience reflection sessions by participants and families applying self-care Facilitate group discussion and questions and answers	Online group session via closed Facebook group	Supporting	Self-report on activities
Session 4 (week 3)	Apply strategies to improve home safety	Shared experiences Review environmental safety and accident prevention through video Facilitate group discussion	Online group session via closed Facebook group	Supporting	Self-report of home changes
Session 5 (week 4)	Use available resources to sustain self-care	Discuss barriers and apply problem-solving strategies Engage family support Reflect on resource use	Online group session via closed Facebook group	Providing an environment that promotes personal development	Family attendance

#### Family Involvement

Family members participated in all intervention sessions delivered through the Facebook group. They received the same educational content as participants and were encouraged to support self-care behaviors, including medication adherence, dietary management, physical activity, and home safety practices. During the sessions, family members were invited to share their experiences and discuss strategies they used to support participants in managing osteoporosis.

#### Facebook Group Privacy Protection

To protect their privacy, participants were asked to create new Facebook accounts exclusively for this study. Participants were instructed to use pseudonyms rather than their real names. Each participant and 1 family member shared the same study-specific account, resulting in 23 Facebook accounts. All live Facebook group sessions were conducted with participants’ cameras turned off, and only pseudonyms were displayed during the sessions to maintain confidentiality.

#### Intervention Fidelity

Intervention fidelity was monitored throughout the study to assess intervention delivery and participant engagement. All intervention sessions were delivered by the principal investigator according to a standardized intervention protocol. Four educational videos covering osteoporosis and medication, nutrition, exercise, and fall prevention were uploaded to the private Facebook group. Facebook analytics recorded 71 views for the osteoporosis and medication video, 165 views for the exercise video, 73 views for the nutrition video, and 90 views for the accident prevention video. Because Facebook did not permit tracking of individual viewing histories or downloads, participant engagement was also assessed through attendance to the weekly live Facebook sessions. All participants attended 1 live session per week, resulting in a 100% (23/23) attendance rate across the 4 scheduled sessions for each participant.

Participants and their family members were asked open-ended questions about the educational content, video viewing experience, attendance to the online sessions, and implementation of self-care activities. Participants were able to accurately describe the educational content and discuss its application to self-care, indicating that all participants had viewed the videos. Any misunderstandings were clarified immediately by the principal investigator during the discussions. All 23 Facebook study accounts completed the intervention, and 3 (13%) participants who missed their originally scheduled sessions attended an alternative session after receiving a reminder from the researcher.

### Outcome

The primary outcome was self-care, which was measured using the Self-Care of Osteoporosis Scale (SCOS) [7], a self-reported instrument.

### Instruments

#### Overview

The SCOS, developed by Cittadini et al [7], consists of 15 items across 3 domains: medication adherence, diet and exercise, and safety to prevent accidents. The scale uses a 5-point Likert scale ranging from “never” (1 point) to “always” (5 points). The total SCOS score was calculated as the mean of the 15 item scores, yielding a possible score ranging from 1 to 5, with higher mean scores indicating better self-care behaviors. The original instrument demonstrated excellent internal consistency, with Cronbach α values of 0.94 for the overall scale, 0.84 for medication adherence, 0.88 for diet and physical activity, and 0.85 for safety performance. As the SCOS had not previously been used in Thailand, permission was obtained from the original instrument developer, and the instrument was translated using a standard forward-backward translation process followed by expert review. In the present study, the Thai version demonstrated acceptable internal consistency at baseline, with a Cronbach α value of 0.93 for the overall scale, 0.56 for medication adherence, 0.80 for diet and physical activity, and 0.70 for safety performance. At the postintervention time point, Cronbach α values were 0.95 for the overall scale, 0.79 for medication adherence, 0.91 for diet and physical activity, and 0.89 for safety performance.

Back pain was assessed using the visual analog scale, a 10-cm line representing pain intensity from 0 to 10, with each centimeter corresponding to 1 point [19].

A process evaluation form was used to assess participants’ engagement in reflective discussions after viewing the videos. Four open-ended questions were used to explore participants’ experiences: (1) weekly viewing experience, (2) most preferred content and reasons, (3) least preferred content and reasons, and (4) suggestions for improving content or presentation.

#### Validity Testing and Tryout

The content validity of the intervention and instruments was evaluated by 5 experts in osteoporosis and physical therapy. The content validity index was 0.86, indicating good content validity. The instruments were also pilot-tested with 30 individuals with osteoporosis who met the inclusion criteria but were not enrolled in the study to assess the clarity and readability of the instruments.

### Unit of Analysis

Although family members participated in the intervention sessions, outcome measures were collected only from participants with osteoporosis. Therefore, the individual participant was the unit of analysis.

### Statistical Analysis

Data were analyzed using SPSS (version 29.0; IBM Corp). Normality was assessed using the Kolmogorov-Smirnov test, and all variables were normally distributed; therefore, parametric tests were applied. Baseline homogeneity between groups was examined using the chi-square test, Fisher exact test, and independent-sample *t* test as appropriate. Differences in overall self-care following the intervention were analyzed using analysis of covariance (ANCOVA), whereas differences in the 3 domains of self-care were examined using multivariate ANCOVA (MANCOVA). Statistical significance was set at *P*<.05. To facilitate transparency and reproducibility, statistical outputs from SPSS, including baseline comparisons and ANCOVA and MANCOVA results, are provided in Multimedia Appendix 2.

Prior to the main analyses, the assumptions underlying ANCOVA and MANCOVA were evaluated. Normality of residuals was assessed using the Shapiro-Wilk and Kolmogorov-Smirnov tests. The Shapiro-Wilk test indicated a slight deviation from normality (*W*=0.946; *P*=.03), whereas the Kolmogorov-Smirnov test indicated no significant departure from normality (*P*=.07). Although the Shapiro-Wilk test suggested a deviation from normality, ANCOVA and MANCOVA are considered robust to moderate violations of normality when group sizes are equal (n=23 per group); therefore, parametric analyses were retained. Linearity between the covariate (baseline self-care score) and the postintervention outcome was confirmed (*F*_1,43_=113.914; *P*<.001). The assumption of homogeneity of regression slopes was evaluated by including a group × baseline self-care mean score interaction term in the ANCOVA model and was satisfied as the interaction was not statistically significant (*F*_1,42_=3.626; *P*=.06). The Levene test indicated that the assumption of homogeneity of variance was satisfied for the overall self-care score (*F*_1,44_=2.989; *P*=.09).

For MANCOVA, the Bartlett test of sphericity confirmed significant intercorrelations among the 3 self-care domains, supporting the use of a multivariate approach (*χ*^2^_3_=47.3; *P*<.001). Pearson correlation coefficients among the 3 postintervention domain scores ranged from 0.739 to 0.861 (*P*<.001 in all cases), indicating no evidence of multicollinearity (*r*<0.90). Homogeneity of variance-covariance matrices, assessed using the Box *M* test, was violated (*M*=21.231; *F*_6_=3.276; *P*=.003). Therefore, the Pillai trace was used to evaluate multivariate group differences because it is robust to violations of the assumption of homogeneity of covariance matrices. However, the baseline mean score for the diet and physical activity domain differed significantly between the groups (*P*=.04), whereas the baseline mean scores for the medication adherence and safety performance domains did not. To account for this baseline difference, the corresponding baseline SCOS domain mean scores were included as covariates in the MANCOVA model. Therefore, the postintervention group comparisons were based on adjusted mean scores after controlling for the corresponding baseline SCOS domain mean scores, minimizing the potential influence of baseline imbalance on the interpretation of the between-group differences.

### Ethical Considerations

Ethics approval was obtained from Chulalongkorn University (022/68) and the Faculty of Medicine of Ramathibodi Hospital (MURA2025/477). All participants provided written informed consent prior to participation. To ensure confidentiality, all data were stored in password-protected files accessible only to the research team. Participants in the intervention group received a stipend of US $8 to offset internet-related expenses associated with participation in the intervention.

## Results

### Homogeneity Test for Participant Characteristics

**Figure 1.**
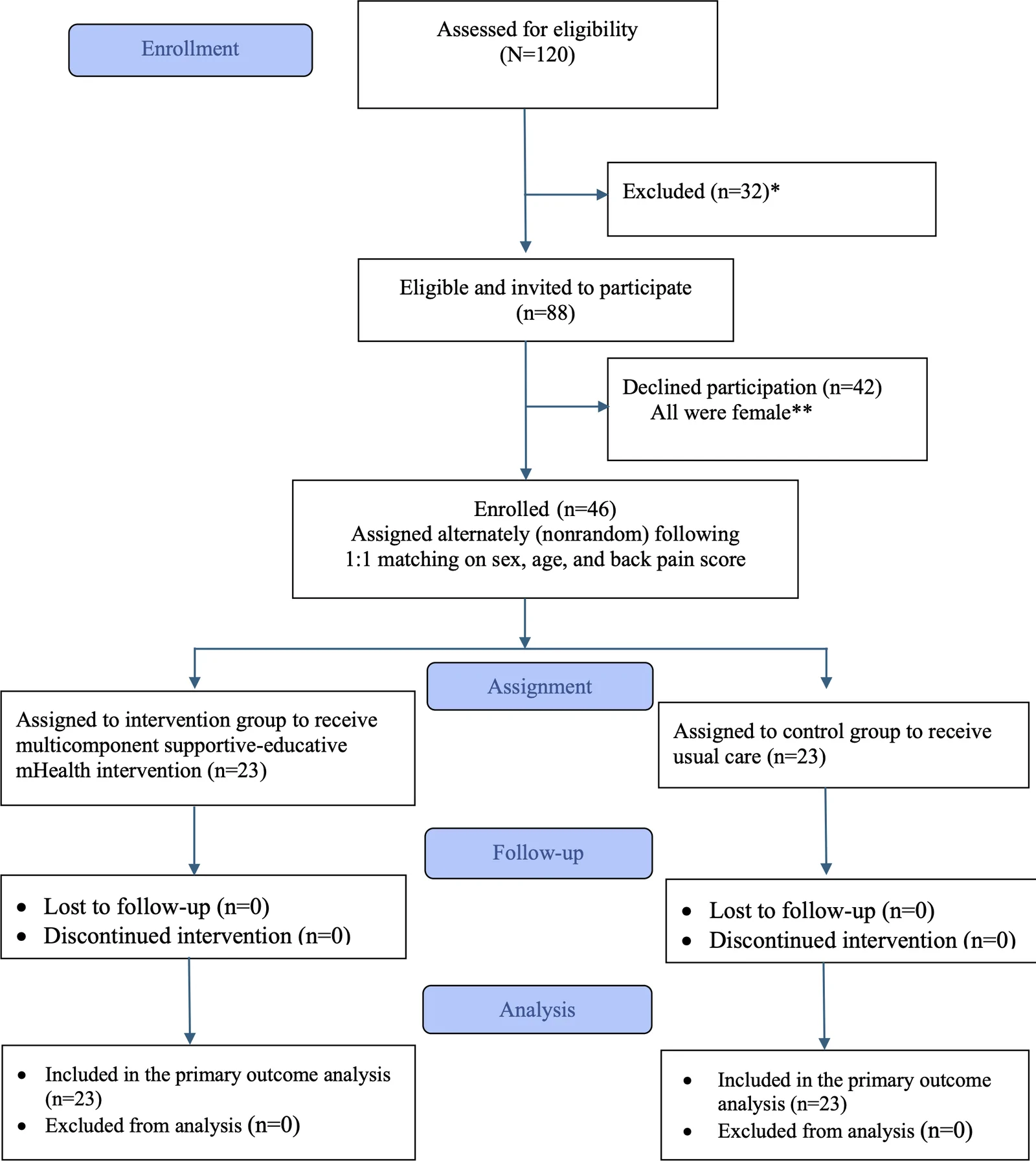
Participant flow diagram. *Reasons for exclusion: severe pain (n=18), scheduled surgery (n=9), and hearing impairments (n=5). **Reasons for declined participation: time constraints or being busy (n=28), family-related inconvenience (eg, relative unavailable; n=9), and having already sought information independently (eg, reading materials or video clips; n=5). Some individuals reported more than one reason for declining participation. mHealth: mobile health.

A total of 46 participants were included in the study, with 23 (50%) in the intervention group and 23 (50%) in the comparison group (Figure 1). No participants dropped out of the program. The test assessing the homogeneity of participant characteristics and dependent variable (self-care) showed no significant differences between the 2 groups, indicating that they were comparable at baseline (Table 2).

**Table 2.** Homogeneity test of participant characteristics and dependent variables.

Characteristics	Intervention group (n=23)	Comparison group (n=23)	*P* value
Sex, n (%)			>.99^a^
Female	20 (87.0)	20 (87.0)	
Male	3 (13.0)	3 (13.0)	
Age (y), mean (SD; range)	71.04 (8.5; 56-88)	70.43 (8.9; 56-87)	.81^b^
Marital status, n (%)			>.99^c^
Single or divorced	4 (17.4)	3 (13.0)	
Married	19 (82.6)	20 (87.0)	
Educational level, n (%)			.23^a^
Secondary school or lower	12 (52.2)	7 (30.4)	
Bachelor’s degree or higher	11 (47.8)	16 (69.6)	
Monthly income (US $), n (%)			.77^a^
≤700	13 (56.5)	14 (60.9)	
>700	10 (43.5)	9 (39.1)	
Health insurance scheme, n (%)			.20^a^
Civil servant medical benefit scheme	18 (78.3)	14 (60.9)	
Self-pay	5 (21.7)	9 (39.1)	
Duration of osteoporosis (y), n (%)			.13^a^
<5	16 (69.6)	11 (47.8)	
5-10	7 (30.4)	12 (52.2)	
Underlying diseases, n (%)			.67^a^
Hypertension	6 (26.1)	5 (21.7)	
Bone and joint diseases	10 (43.5)	13 (56.5)	
Others	7 (30.4)	5 (21.7)	
BMI (kg/m^2^), mean (SD; range)	22.81 (3.48; 18.02-33.33)	23.24 (3.32; 17.48‐33.31)	

a Chi-square test.

b Independent-sample *t* test.

c Fisher exact test.

### Between-Group Differences in Postintervention Self-Care

ANCOVA was conducted to compare postintervention self-care mean scores between the intervention and control groups while controlling for baseline self-care mean scores. The results showed that the intervention group had significantly higher postintervention self-care mean scores than the control group (adjusted mean difference 0.97, 95% CI 0.88-1.06; *F*_1,43_=459.29; *P*<.001; partial η^2^=0.914; Table 3).

MANCOVA was conducted to examine differences in the 3 domains of self-care between the intervention and comparison groups after controlling for baseline domain mean scores (medication adherence, diet and physical activity, and safety performance). The overall multivariate group difference was statistically significant, as indicated by the Pillai trace (*V*=0.939; *F*_3,39_=198.45; *P*<.001; partial η^2^=0.939), suggesting significant differences between groups for all 3 domains after controlling for baseline domain mean scores. Compared with the comparison group, the intervention group demonstrated significantly higher postintervention mean scores for diet and physical activity (adjusted mean difference 1.02, 95% CI 0.89-1.15; *F*_1,41_=245.04; *P*<.001; partial η^2^=0.857), safety performance (adjusted mean difference 1.04, 95% CI 0.88-1.20; *F*_1,41_=171.20; *P*<.001; partial η^2^=0.807), and medication adherence (adjusted mean difference 0.81, 95% CI 0.68-0.95; *F*_1,41_=144.54; *P*<.001; partial η^2^=0.779; Table 3).

**Table 3.** Results of analysis of covariance (ANCOVA) and multivariate ANCOVA (MANCOVA) comparing postintervention outcomes between groups after controlling for baseline mean scores.

Domains	Baseline (covariate), raw mean (SD)			Postintervention time point, raw mean (SD)		Postintervention time point, adjusted mean (SE)		Adjusted mean difference (95% CI)	*F* test (*df*)	*P* value	Effect size, partial η^2^
	Intervention group	Control group	*P* value	Intervention group	Control group	Intervention group	Control group				
ANCOVA											
Total mean score (1-5)	3.18 (0.59)	2.94 (0.28)	.10^a^	4.20 (0.33)	3.12 (0.22)	4.14 (0.03)	3.18 (0.03)	0.97^b^ (0.88-1.06)	459.29 (1,43)	<.001^c^	0.914
MANCOVA (Pillai trace=0.939)									198.45 (3,39)	<.001^c^	0.939
Medication adherence (range 1-5)	3.16 (0.86)	3.03 (0.45)	.52^a^	4.23 (0.44)	3.33 (0.30)	4.19 (0.05)	3.38 (0.05)	0.81^b^ (0.68-0.95)	144.54 (1,41)	<.001^c^	0.779
Diet and physical activity (range 1-5)	3.22 (0.69)	2.89 (0.28)	.04^a^^,c^	4.17 (0.34)	3.02 (0.28)	4.11 (0.05)	3.09 (0.05)	1.02^b^ (0.89-1.15)	245.04 (1,41)	<.001^c^	0.857
Safety performance (range 1-5)	3.13 (0.69)	2.97 (0.42)	.36^a^	4.23 (0.46)	3.11 (0.32)	4.19 (0.06)	3.16 (0.06)	1.04^b^ (0.88-1.20)	171.20 (1,41)	<.001^c^	0.807

a Independent-sample *t* test.

b Difference calculated as the adjusted mean of the intervention group minus that of the comparison group.

c Statistically significant (*P*<.05).

## Discussion

### Main Outcomes

The findings indicate that the multicomponent supportive-educative mHealth intervention was associated with higher postintervention self-care mean scores among individuals with osteoporosis. Higher adjusted mean scores were observed across all domains of self-care (medication adherence, diet and physical activity, and safety performance), with the largest between-group difference observed for diet and physical activity.

### Interpretation

The observed between-group difference may reflect the combined contribution of several intervention components. These components included the provision of clear and reliable disease-related information, weekly nurse-participant interactions, family support, sustained attention, and digital reinforcement. Disease-related knowledge may support self-care by enhancing individuals’ understanding of their condition and increasing their confidence in managing it. This is consistent with the supportive-educative nursing system by Orem et al [13], which emphasizes teaching and guidance to strengthen self-care agency. In addition, the weekly nurse-participant interactions provided opportunities for participants to discuss concerns, receive feedback, and clarify misunderstandings, whereas family involvement and digital reinforcement may have strengthened self-care behaviors and sustained participants’ engagement throughout the intervention.

Family support may also have played a role in these observed differences. When family members learn alongside individuals with osteoporosis, this facilitates shared review and reinforcement of the content and supports engagement in self-care. This reflects the emphasis by Orem et al [13] on providing an environment that supports personal development and self-care. Although evidence on family support intervention for osteoporosis management is limited, findings from exploratory studies suggest that family involvement may support self-care [17]. When one person misunderstands or forgets information, others can provide reminders or exchange ideas, thereby supporting sustained self-care practices. In many countries, including Thailand, extended family living and close family ties are common.

Although higher postintervention self-care mean scores were observed in the intervention group than in the control group, both groups showed increases in self-care mean scores over time. Therefore, the observed between-group differences should be interpreted cautiously as factors other than the intervention may also have influenced the findings. Participants in the control group received usual care, and repeated completion of the self-care assessments may have increased awareness of self-care behaviors. To reduce potential confounding, participants were matched on sex, age, and back pain score, factors previously associated with self-care [6,9]. In addition, baseline self-care mean scores were controlled for in the ANCOVA and MANCOVA analyses. However, the results should be interpreted as indicating an association between the multicomponent supportive-educative mHealth intervention and higher postintervention self-care mean scores rather than as definitive evidence of a causal effect.

The multicomponent supportive-educative intervention delivered partly through Facebook provided accessible and flexible learning. Video-based content allowed for repeated viewing, whereas remote interaction with nurses enabled timely guidance and reinforcement. This continuous connection supported engagement beyond clinical settings and facilitated shared learning with family members. However, this finding contrasts with those of previous studies of app-based interventions that found no significant differences on physical activity, calcium intake, or vitamin D intake, possibly because of the lack of a personalized and interactive approach [16].

Participants’ experience provides insights into how the intervention influenced self-care. Participants found dietary guidance easy to understand and reported actively implementing fall prevention strategies; in contrast, exercise was less frequently adopted. In this study, both groups reported rarely engaging in exercise before the intervention. After the intervention, however, the intervention group reported sometimes engaging in exercise, whereas the control group continued to report rarely engaging in exercise. Although exercise can increase bone mineral density, enhance muscle strength, and improve body balance, thereby reducing the risk of falls, adherence remains challenging. Previous studies have reported that exercise adherence among individuals with osteoporosis varies from 59% to 100% even when expert coaching or accessible facilities are provided [20]. These findings highlight the need for additional strategies to promote exercise adherence.

### Implications for Practice and Research

This study has implications for practice. The observed between-group differences suggest that the multicomponent supportive-educative intervention delivered partly through Facebook may warrant evaluation in randomized, attention-controlled studies before clinical implementation. Nurses may consider using digital platforms to extend care beyond clinical settings and engage family members as active partners in care while recognizing that the effectiveness of this approach requires confirmation in more rigorous studies.

### Limitations

This study has several limitations. As an exploratory pilot study with nonrandom allocation, the findings should be interpreted with caution because factors other than the intervention, including the unequal intensity of support between groups, may have been associated with the observed between-group differences. The substantial effect sizes should also be interpreted cautiously as the relatively small sample size and reliance on self-reported measures may have influenced the magnitude of the estimated associations. In addition, recruitment from a single hospital may limit the generalizability of the findings. The medication adherence domain demonstrated low internal consistency at baseline (Cronbach α=0.56), which may have reduced the precision of the estimated associations for this domain. Self-care was assessed using self-reported measures, which may be subject to response bias. Participants and researchers were not blinded to group assignment, and contamination between groups may have occurred because participants were recruited from the same setting. Finally, the short follow-up period did not allow for evaluation of the long-term sustainability of the observed improvements. Future studies using randomized controlled designs, larger and more diverse samples, objective measures of self-care, and longer follow-up periods are needed to confirm these findings.

### Conclusions

In conclusion, the multicomponent supportive-educative mHealth intervention was associated with higher self-care mean scores across multiple domains among individuals with osteoporosis. The observed between-group differences may reflect the integration of educational content, family involvement, ongoing support, and digital delivery. However, they should be interpreted with caution given the exploratory pilot study and relatively small sample size. Further rigorous randomized controlled trials are needed to confirm the efficacy of this intervention.

## Supplementary material

10.2196/101025 Multimedia Appendix 1 Intervention protocol.

10.2196/101025 Multimedia Appendix 2 Analysis of covariance and multivariate analysis of covariance statistical output.

10.2196/101025 Checklist 1 TREND statement checklist.

## Abbreviations

ANCOVA: analysis of covariance
MANCOVA: multivariate analysis of covariance
mHealth: mobile health
SCOS: Self-Care of Osteoporosis Scale
TREND: Transparent Reporting of Evaluations With Nonrandomized Designs
