# Supplementary material for: Multicomponent Supportive-Educative mHealth Intervention for Self-Care Among Individuals With Osteoporosis in Thailand: Exploratory Pretest-Posttest Pilot Study
Source: JMIR Nurs. 2026 Aug 20;9:e101025. doi: 10.2196/101025 (PMC13492480; doi:10.2196/101025)
Supplement: Supplementary file 2 [file nursing-v9-e101025-s001.docx]

**Appendix 1. Intervention Protocol**

**Study Title**

Supportive-Educative mHealth Intervention on Self-care among Individuals with Osteoporosis in Thailand: An Exploratory Pilot Study

**Objectives**

To compare self-care among individuals with osteoporosis who received a supportive-educative mHealth intervention with those who received usual care.

**Study Design**

The study design is exploratory pilot study using pretest–posttest design with a non-equivalent control group.

**Setting**

A university hospital in Bangkok Thailand, between July and August 2025. The intervention was delivered over four weeks (28 days).

**Participants**

***Inclusion Criteria***

Participants were eligible if they:

1. were aged 50 years or older;
2. had received osteoporosis treatment for at least 6 months;
3. lived with family members or relatives; and
4. had access to Facebook through either themselves or a family member.

***Exclusion Criteria***

Participants were excluded if they:

1. were severely ill;
2. were scheduled for surgery during the intervention period;
3. had speech or hearing impairment; or
4. had cognitive impairment, defined as a score ≤22 on the Thai version of the Mini-Mental State Examination (MMSE).

**Group Assignment**

Participants were assigned alternately to the intervention and comparison groups in a 1:1 ratio. Assignment was used to maintain comparable group sizes and facilitate matching based on sex, age, and back pain score, which were considered potential factors associated with self-care.

**Intervention Procedures**

***1. Baseline visit (Week 0)***

Participants in both the intervention and comparison groups met the researcher during their outpatient clinic visit. Baseline self-care was assessed using the Self-Care of Osteoporosis Scale (SCOS). Participants in the intervention group received a printed handbook containing the same educational content as the four educational videos and were introduced to the intervention procedures. They also received a stipend of USD 8 to offset internet-related expenses associated with participation. Participants in the comparison group received routine nursing education focusing on medication, diet, and exercise. This education was delivered verbally without written educational materials or exercise demonstration videos. Participants in both groups could confirm or reschedule appointments through the hospital application.

***2. Facebook Account Setup***

A new Facebook account was created exclusively for this study. Participants created a study-specific Facebook account using a pseudonym rather than their personal account. Each participant and one family member shared the same study-specific account, resulting in 23 Facebook accounts.

***3. Educational materials (Week 1)***

On the first day of Week 1, four educational videos (approximately 10 minutes each) were uploaded to the private Facebook group. The videos covered:

1. Osteoporosis and medication
2. Exercise
3. Diet
4. Safety performance

The videos remained available throughout the 4-week intervention. Participants also received a printed handbook containing the same educational content.

***4. Weekly Facebook Live sessions (Weeks 1–4)***

Throughout the 4-week intervention, Live Facebook sessions were conducted daily between 3:00 PM and 4:00 PM. Participants attended one 15-minute session per week in small groups of three to four study accounts. Sessions were scheduled over seven days to accommodate all participants. Following the scheduled 15-minute discussion, the researcher remained online until 4:00 PM to answer additional questions.

Participants who missed their scheduled session received a reminder through Facebook Messenger and attended an alternative session. All live sessions were conducted with participants' cameras turned off, and only pseudonyms were displayed to maintain confidentiality.

During each session, the researcher asked open-ended questions about the educational content, participants' experiences applying the recommendations, and any challenges encountered. Participants' understanding was reviewed, misunderstandings were clarified, and individualized feedback was provided.

The weekly themes were as follows:

Week 1: Osteoporosis, medication adherence, diet, and exercise. Participants discussed the educational videos and their understanding of the content.

Week 2: Experiences of applying knowledge to self-care practices.

Week 3: Home safety, accident prevention, and sharing experiences of implementing self-care practices.

Week 4: Barriers to self-care, problem-solving strategies, family support, and reflection on the use of available resources to support self-care.

***5. Follow-up Assessment (Week 5)***

During Week 5, participants in both groups attended a second outpatient clinic visit. Self-care was reassessed using the SCOS.

***6. Completion of the Study***

After completing the follow-up assessment, the researcher thanked all participants for their participation. Participants in the comparison group received the printed handbook containing the same educational content as the educational videos as appreciation for their participation.

**Outcomes and Measures**

The primary outcome was self-care, measured using the Self-Care of Osteoporosis Scale (SCOS).

**Ethics**

Ethical approval was obtained from Chulalongkorn University (No. 022/68) and Faculty of Medicine Ramathibodi Hospital (MURA2025/477). All participants provided written informed consent prior to participation. To ensure confidentiality, all data were stored in password-protected files accessible only to the research team.

-----------------
