# Supplementary material for: Multicomponent Supportive-Educative mHealth Intervention for Self-Care Among Individuals With Osteoporosis in Thailand: Exploratory Pretest-Posttest Pilot Study
Source: JMIR Nurs. 2026 Aug 20;9:e101025. doi: 10.2196/101025 (PMC13492480; doi:10.2196/101025)
Supplement: Supplementary file 3 [file nursing-v9-e101025-s002.docx]

**Appendix 2. ANCOVA and MANCOVA Statistical Output**

**1. Self-care at Baseline**

**2. Self-care at Postintervention (Raw means)**

**3. Self-care at Postintervention (Adjusted means)**

**4. Comparison of Adjusted Mean Postintervention Total Self-Care Scores Between Intervention and Comparison Groups (ANCOVA)**

**5. Comparison of Adjusted Mean Postintervention Self-Care Domain Scores Between the Intervention and Comparison Groups (MANCOVA)**
